# Supplementary material for: Association between oral microbiome and seven types of cancers in East Asian population: a two-sample Mendelian randomization analysis
Source: Front Mol Biosci. 2023 Nov 21;10:1327893. doi: 10.3389/fmolb.2023.1327893 (PMC10702768; doi:10.3389/fmolb.2023.1327893)
Supplement: Supplementary file 1 [file DataSheet1.ZIP › Supplementary Materials/MR plots for tongue/tongue═╝/Gastric cancer/pheno.229_to_gastric cancer_forest.pdf]

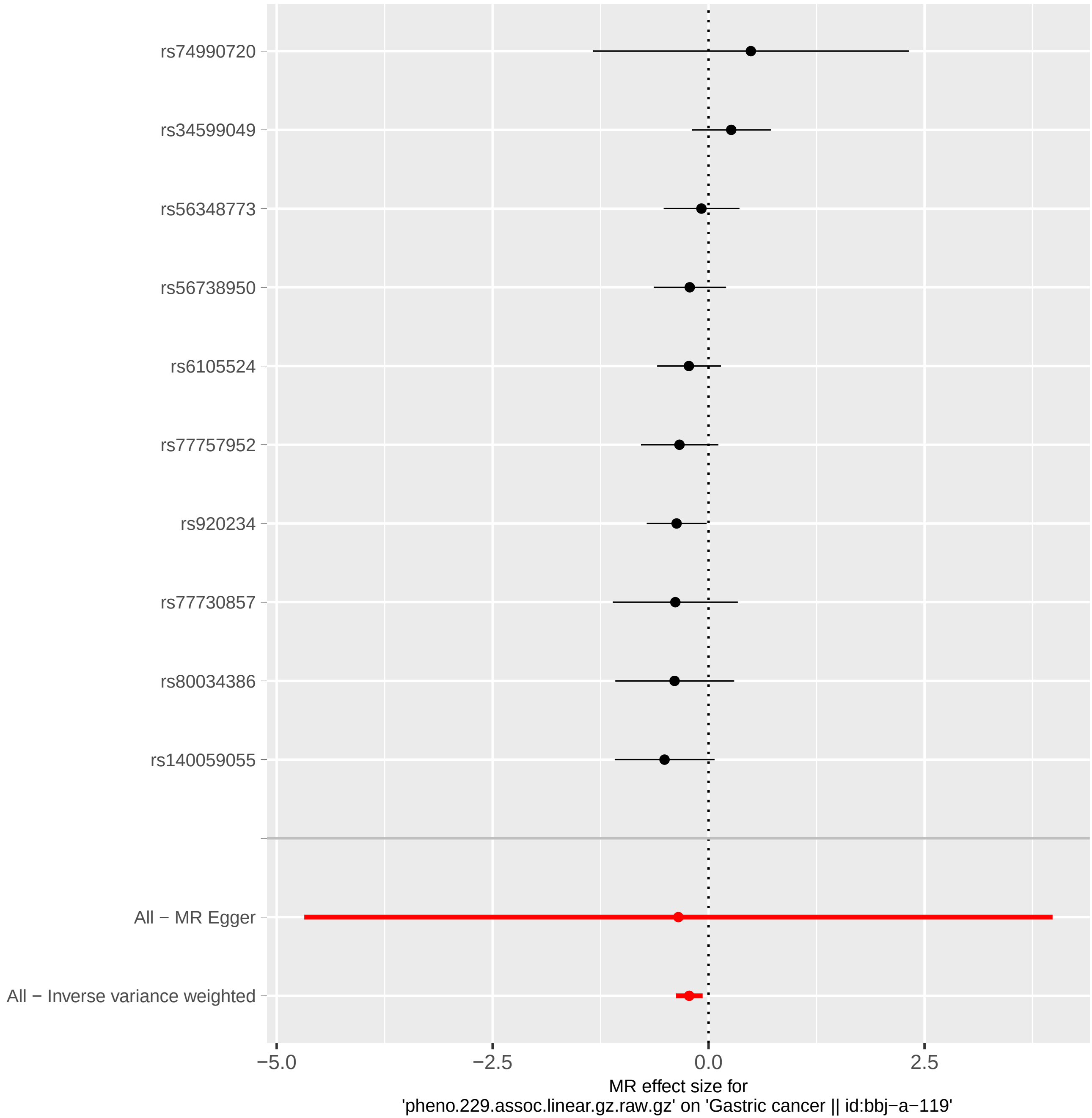
rs74990720
rs34599049
rs56348773
rs56738950
rs6105524
rs77757952
rs920234
rs77730857
rs80034386
rs140059055
All − MR Egger
All − Inverse variance weighted
−5.0
−2.5
0.0
2.5
MR effect size for
'pheno.229.assoc.linear.gz.raw.gz' on 'Gastric cancer || id:bbj−a−119'
